# Unveiling Phenotypic Heterogeneity in Coronary Spastic Angina Through Multidimensional Risk Profiling: The FUJI-SPASM Study

**DOI:** 10.1016/j.jacadv.2026.102628

**Published:** 2026-03-25

**Authors:** Takamitsu Nakamura, Tuan Hoang Nguyen, Takeo Horikoshi, Juntaro Deyama, Manabu Uematsu, Toru Yoshizaki, Tsuyoshi Kobayashi, Kazuto Nakamura, Akira Sato

**Affiliations:** Department of Cardiovascular Medicine, University of Yamanashi Faculty of Medicine, Chuo, Japan

**Keywords:** acetylcholine test, coronary spasm, endothelial dysfunction, inflammatory phenotype, metabolic phenotype, principal component analysis

## Abstract

**Background:**

Coronary spastic angina (CSA) is a functional coronary disorder causing recurrent ischemia without significant atherosclerosis, but the interaction between metabolic and inflammatory abnormalities remains unclear.

**Objectives:**

The purpose of this study was to identify clinically meaningful CSA phenotypes based on metabolic and inflammatory profiles and to determine phenotype-specific determinants of coronary spasm and endothelial dysfunction.

**Methods:**

We analyzed 568 patients with suspected CSA and no obstructive coronary artery disease from the FUJI-SPASM (Feature-based Understanding of Joint Investigation for coronary Spasm Phenotypes and Associated Stratified Mechanisms) registry. Coronary spasm was assessed using intracoronary acetylcholine provocation testing. Unsupervised k-means clustering was performed using 20 standardized clinical and biochemical variables. Phenotype-specific determinants of spasm were identified using adaptive least absolute shrinkage and selection operator regression followed by multivariable logistic regression within each cluster. Endothelial function was assessed by flow-mediated dilation in a subset of 159 patients.

**Results:**

Two phenotypes were identified. Cluster 0 (n = 231) showed metabolically dominant profiles with atherogenic dyslipidemia and insulin resistance, whereas cluster 1 (n = 337) showed inflammation-dominant profiles with preserved lipid levels but elevated high-sensitivity C-reactive protein (hs-CRP). Lower high-density lipoprotein cholesterol (HDL-C) was associated with coronary spasm in both clusters, while higher hs-CRP was independently associated with spasm only in the inflammation-dominant cluster. An HDL-C/hs-CRP–based stratification demonstrated a graded increase in spasm prevalence and worsening endothelial function.

**Conclusions:**

CSA comprises distinct metabolic and inflammatory phenotypes with differing determinants of coronary spasm and endothelial dysfunction. Reduced HDL-C represents a shared vulnerability across phenotypes, while inflammation confers additional risk. HDL-C/hs-CRP–based stratification provides a clinically accessible framework for individualized CSA risk assessment.

Coronary spastic angina (CSA) is a clinical syndrome characterized by transient coronary artery constriction that can induce myocardial ischemia, arrhythmias, or sudden cardiac death, even in the absence of obstructive coronary artery disease.^1^^,^^2^ Two principal mechanisms have been implicated in the pathogenesis of coronary artery spasm (CAS): endothelial dysfunction, which impairs nitric oxide–mediated vasodilation, and hypercontractility of vascular smooth muscle cells, often mediated by enhanced Rho-kinase activity.^3^^,^^4^ These mechanisms are not mutually exclusive and may coexist in varying proportions among individuals. Importantly, recent studies suggest that individual variability in the dominance of these mechanisms may underlie differences in clinical expression, treatment response, and long-term outcomes,^5^, ^6^, ^7^ underscoring the need for patient-specific phenotypic assessments.

Calcium-channel blockers (CCBs) are widely recognized as the cornerstone therapy for CSA, and their use has been shown to improve the prognosis and reduce the incidence of major adverse cardiac events.^1^^,^^2^^,^^8^ However, accumulating data indicate that pharmacologic treatment does not fully suppress recurrent anginal symptoms in a substantial proportion of patients. For example, over 60% of patients continue to experience anginal attacks despite long-term CCB therapy, and nearly 40% report more than one episode per month.^9^ These persistent symptoms highlight the limitations of current therapeutic strategies and the pressing need to better understand the biological heterogeneity underlying CSA.

Clinical heterogeneity of CSA has been increasingly recognized. Some patients display metabolic abnormalities, such as insulin resistance and dyslipidemia,^10^ whereas others exhibit elevated inflammatory markers despite having relatively normal lipid profiles.^11^ These divergent presentations suggest that CSA encompasses multiple biological phenotypes driven by distinct pathophysiological processes. However, the systematic classification and mechanistic linkage between these clinical phenotypes and CAS susceptibility remain poorly defined.

Recent advances in data-driven analytical approaches have enabled the identification of clinically meaningful phenotypes in complex cardiovascular conditions. Unsupervised clustering methods, in particular, allow patient stratification based on multidimensional clinical and biochemical profiles without prespecified assumptions, thereby facilitating the exploration of heterogeneity in disease mechanisms and vasomotor dysfunction.^12^^,^^13^

Both metabolic and inflammatory stresses have been shown to contribute to CAS through distinct mechanisms, either by impairing endothelial function or promoting smooth muscle hypercontractility.^13^^,^^14^ In our previous optical coherence tomography studies, we also demonstrated that CAS is associated with preserved medial thickness, even in the presence of intimal thickening.^14^^,^^15^ In contrast, arteries without spasms often exhibit medial thinning in the setting of advanced intimal hyperplasia.^16^, ^17^, ^18^ These findings suggest that a specific structural and functional arterial milieu—combining medial integrity with moderate intimal changes—may be a prerequisite for the manifestation of CAS. Understanding how metabolic and inflammatory alterations influence these vascular characteristics may yield deeper insights into CAS pathophysiology.

To date, no study has comprehensively classified CSA phenotypes based on both metabolic and inflammatory parameters while linking these profiles to coronary vasomotor reactivity and endothelial function in patients receiving standard pharmacological therapy. The present study aimed to address this gap by applying an unsupervised clustering approach to multidimensional biochemical and clinical data, in order to identify biologically distinct phenotypes of CSA and to examine their associations with spasm susceptibility and vascular dysfunction.

## Methods

### Study patients

**Figure 1:**
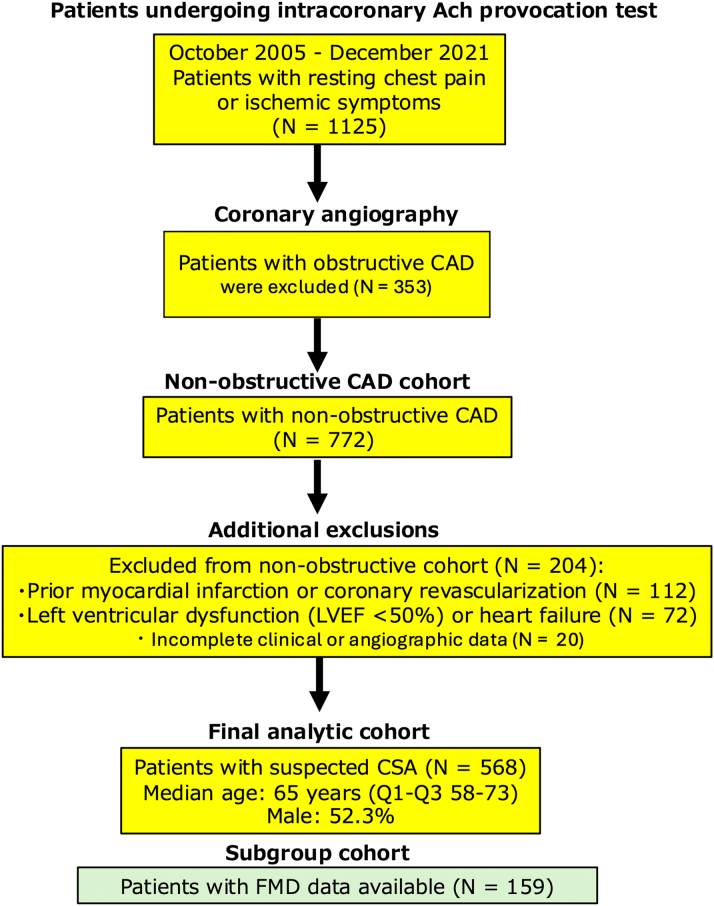
**Study Design and Patient Flow** Among 1,125 patients who underwent intracoronary acetylcholine provocation testing, those with obstructive coronary artery disease were excluded based on coronary angiography. Additional exclusions were applied from the nonobstructive cohort according to predefined criteria. When multiple exclusion criteria were present, each patient was assigned to a single exclusion category. ACh = acetylcholine; CAD = coronary artery disease; CSA = coronary spastic angina; FMD = flow-mediated dilation; LVEF = left ventricular ejection fraction.

This study was conducted as part of the FUJI-SPASM Study (Feature-based Understanding of Joint Investigation for coronary Spasm Phenotypes and Associated Stratified Mechanisms), a single-center, observational project based on the Cardiovascular Disease Database at the University of Yamanashi Hospital (UMIN000058137). Between October 2005 and December 2021, 1,125 patients who presented with resting chest pain or related ischemic symptoms underwent intracoronary acetylcholine (ACh) provocation testing (Figure 1). Coronary angiography was performed in all patients to exclude obstructive coronary artery disease, and patients with ≥50% stenosis in any major coronary artery were excluded. Of these, 772 patients without significant coronary artery stenosis were included. Patients were excluded if they had a left ventricular ejection fraction <50%, a history of heart failure, prior myocardial infarction, prior coronary revascularization, or incomplete clinical or angiographic data. All exclusions were applied prior to the analytic cohort definition. When patients met more than one exclusion criterion, they were assigned to a single exclusion category based on a predefined hierarchical order (prior myocardial infarction or coronary revascularization, followed by left ventricular dysfunction or heart failure, and incomplete data) to avoid double counting. Written informed consent was obtained from all participants prior to the procedures. The study protocol was approved by the Ethics Committee of the University of Yamanashi Hospital (IRB approval numbers: 213 and 1317) and adhered to the principles of the 1975 Declaration of Helsinki (2013 revision).

### Laboratory assessment of metabolic and inflammatory profiles

Fasting venous blood samples were collected in the morning or on the day prior to the ACh provocation test. All assays were performed at the central clinical laboratory of the University of Yamanashi Hospital under standardized quality-controlled conditions. The lipid profiles included total cholesterol, triglycerides, high-density lipoprotein (HDL) cholesterol (HDL-C), low-density lipoprotein (LDL) cholesterol (LDL-C) (measured and calculated), and non–HDL-C. Glycemic indices, including fasting glucose, insulin, glycated hemoglobin A1c, and insulin resistance were estimated using homeostasis model assessment-insulin resistance (HOMA-IR): (fasting insulin × fasting glucose)/405. Free fatty acids (FFA) were assessed using an enzymatic colorimetric assay. Inflammatory markers included high-sensitivity C-reactive protein (hs-CRP) and plasma fibrinogen, which were measured using a nephelometric immunoassay and the Clauss method, respectively. Apolipoproteins (ApoA1, ApoA2, ApoB, ApoC2, ApoC3, and ApoE) were measured using immunoturbidimetric assays with commercial kits (Sekisui Medical Co, Ltd).

### Assessment of coronary artery spasm

The details of the procedure have been described previously.^14^^,^^15^ ACh provocation testing was performed according to the Japanese Circulation Society guidelines and prior institutional protocols.^19^ After diagnostic coronary angiography via radial or femoral access, 50 and 100 μg of ACh was injected sequentially into the left coronary artery over 30 seconds each, followed by coronary angiography 1 minute after each injection. A dose (50 μg) was injected into the right coronary artery. CAS positivity was defined as either^1^ transient total or subtotal occlusion with delayed contrast washout or^2^ ≥90% focal narrowing accompanied by typical chest pain and ischemic electrocardiogram (ECG) changes. Continuous ECG and hemodynamic monitoring were performed throughout the procedure. Intracoronary nitrates were administered in cases of prolonged spasm, significant hypotension, arrhythmia, or patient refusal; testing of the contralateral artery was omitted in such cases. Coronary spasm morphology was classified as focal or diffuse according to established angiographic criteria during ACh provocation testing. Focal spasm was defined as a discrete, localized transient luminal narrowing ≥90% occurring within a single coronary segment. Diffuse spasm was defined as continuous or segmental vasoconstriction ≥90% involving two or more adjacent coronary segments or extending over a length of ≥20 mm. In cases of subtotal (≥99%) or total occlusion, spasm morphology was determined based on the longitudinal extent of vasoconstriction observed immediately before complete occlusion or after intracoronary nitrate administration. CCBs and nitrates, except for sublingual nitroglycerin, were withheld for at least 48 hours before testing.

### Measurement of endothelial function

A predefined subset of 159 patients was selected consecutively from the eligible registry population during the study period when flow-mediated dilation (FMD) measurement was routinely integrated into the clinical workflow. The availability of FMD data was determined primarily by logistical and temporal factors related to equipment availability and operator scheduling, rather than by patient risk profile or angiographic findings (Supplemental Table 1). Brachial artery FMD was assessed using high-resolution ultrasonography (7.5-MHz linear-array probe; HP-5500, Philips) per validated protocol.^20^^,^^21^ The artery was imaged longitudinally, 1 to 5 cm proximal to the antecubital fossa, after a 10-minute supine rest. A cuff was inflated on the forearm to 50 mm Hg above systolic pressure for 5 minutes to induce reactive hyperemia, followed by rapid deflation. The arterial diameter was recorded continuously for 2 minutes after deflation. FMD was expressed as the percentage change in vessel diameter from the baseline. Images were analyzed offline by 2 experienced cardiologists (T.H. and M.U.), each with over 10 years of experience in vascular imaging. Reproducibility values (interobserver and intraobserver variability: 0.05 ± 0.03 mm and 0.05 ± 0.02 mm, respectively) were reported in a prior study.

### Clustering and statistical analysis

Clustering is an unsupervised machine learning technique applied to data to detect hidden patterns without the need for a target. A K-means++ algorithm with 300 iterations was performed using 20 standardized variables: lipid markers (LDL-C, HDL-C, triglycerides, non-HDL-C, and FFA), apolipoproteins (ApoA1, ApoA2, ApoB, ApoC2, ApoC3, and ApoE), inflammatory and hemostatic markers (CRP and fibrinogen), glycemic indices (HOMA-IR and hemoglobin A1c), and clinical parameters (age, sex, body mass index, smoking status, and hypertension). Smoking status was categorized as current smoker vs nonsmoker (never or former). Hypertension was defined as systolic blood pressure (BP) ≥140 mm Hg, diastolic BP ≥90 mm Hg, or use of antihypertensive medications. All variables were *z*-score standardized to ensure comparability and equal weighting in the clustering and principal components analysis algorithms. The number of clusters was determined using silhouette coefficients and silhouette analysis plots. Reproducibility of clustering was then assessed in the population excluding outliers identified by the silhouette plot, after dimensionality reduction using principal component analysis. Multivariable logistic regression analysis was used to identify factors associated with each cluster or CAS positivity. Multicollinearity was assessed using the uncentered variance inflation factor, and the least absolute shrinkage and selection operator with adaptive method were evaluated before proceeding with the multivariable logistic regression analysis. The cutoff point determined by the Youden index was then applied to significant associated factors to stratify patients. In the FMD-assessed subgroup, endothelial function was analyzed across groups using one-way analysis of variance (ANOVA) followed by Bonferroni-adjusted post hoc tests.

The distribution of continuous variables was evaluated using the Kolmogorov-Smirnov test and quantile-quantile plots. Continuous variables are presented as median with 25th-75th percentiles (Q1-Q3) or mean ± SD, and categorical variables as counts with percentages. Statistical comparisons were performed using the unpaired *t*-test, Mann-Whitney *U* test, Kruskal-Wallis test, one-way ANOVA, chi-squared or Fisher exact test, as appropriate. Models are presented as OR with 95% CI. All analyses were conducted using STATA version 16.0 (StataCorp, 2019) and Python version 6.5.4 (Python Software Foundation, 2023) with the pandas, scikit-learn, scipy, and statsmodels libraries. Statistical significance was defined as a 2-sided *P* value <0.05.

## Results

### Baseline characteristics and coronary spasm positivity

A total of 568 patients with suspected CSA (median age 65 years [Q1-Q3 58-73], 52.3% male) were included in the analysis. Baseline clinical characteristics of these patients are summarized in Table 1. ACh-provocation testing identified CAS in 242 patients (42.5%). Among the CAS-positive cases, 71 patients (29.3%) exhibited transient total occlusion, 126 (52.1%) showed 99% focal stenosis with delayed contrast washout, and 45 (18.6%) fulfilled the definition of CAS positivity based on 90% to 99% luminal narrowing accompanied by typical chest pain and ischemic ECG changes.

**Table 1:** Comparisons of Baseline Clinical Characteristics of the Study Patients Between Clusters 0 and 1

	Total (N = 568)	Cluster 0 (n = 231)	Cluster 1 (n = 337)	*P* Value
Age (y)	66.5 [58.0-73.0]	65.0 [56.0-72.0]	67.0 [59.0-75.0]	0.006
Male, n (%)	304 (53.5)	119 (51.5)	185 (54.9)	0.43
Smoking, n (%)	244 (43.0)	83 (35.9)	161 (47.8)	0.005
Hypertension, n (%)	296 (52.1)	136 (58.9)	160 (47.5)	0.008
BMI (kg/m^2^)	23.4 [21.7-25.5]	24.0 [22.3-26.0]	23.1 [21.1-25.1]	<0.001
LDL-C (mg/dL)	116 [93-139]	140 [122-158]	102 [84-120]	<0.001
Triglyceride (mg/dL)	121 [87-163]	162 [125-221]	99 [77-127]	<0.001
HDL-C (mg/dL)	47 [40-58]	44 [37-52]	50 [42-60]	<0.001
Non-HDL-C (mg/dL)	141 [117-168]	173 [159-191]	121 [107-140]	<0.001
ApoA1 (mg/dL)	129 [114-145]	124 [109-139]	133 [119-147]	<0.001
ApoA2 (mg/dL)	23 [20-27]	21 [18-24]	24 [22-28]	<0.001
ApoB (mg/dL)	95 [80-112]	113 [103-124]	84 [73-94]	<0.001
ApoC2 (mg/dL)	4.4 [3.3-5.7]	5.70 [4.5-6.9]	3.6 [2.6-4.5]	<0.001
ApoC3 (mg/dL)	9.0 [7.4-11.1]	11.1 [9.2-13.2]	8.30 [6.7-9.4]	<0.001
ApoE (mg/dL)	4.4 [3.7-5.3]	5.3 [4.7-6.0]	4.0 [3.4-4.5]	<0.001
FFA (mg/dL)	434 [312-565]	462 [338-602]	409 [289-537]	<0.001
HOMA-IR	1.38 [0.95-2.19]	1.67 [1.13-2.56]	1.29 [0.86-1.82]	<0.001
HbA1c (%)	5.6 [5.2-6.1]	5.6 [5.3-6.1]	5.5 [5.2-6.0]	0.006
FIB (mg/dL)	304 [262-360]	276 [239-308]	331 [280-389]	<0.001
hs-CRP (mg/dL)	0.2 [0.12-0.39]	0.1 [0.03-0.30]	0.23 [0.14-0.40]	<0.001
Medication use, n (%)				
ACE-I/ARB	87 (15.3)	38 (16.5)	49 (14.5)	0.54
CCB	85 (15.0)	43 (18.6)	42 (12.5)	0.043
Statin	120 (21.1)	57 (24.7)	63 (18.7)	0.086
Aspirin	72 (12.7)	30 (13.0)	35 (10.4)	0.34
Beta-blocker	12 (2.1)	5 (2.2)	7 (2.1)	0.94

Values are median [25th-75th percentiles] or n (%). Hypertension was defined as blood pressure >140/90 mm Hg or use of antihypertensive medication.

ACE-I = angiotensin-converting enzyme inhibitor; Apo = apolipoprotein; ARB = angiotensin II receptor blocker; BMI = body mass index; CCB = calcium-channel blocker; FFA = free fatty acid; FIB = fibrinogen; HbA1c = hemoglobin A1c; LDL-C = low-density lipoprotein cholesterol; HDL-C = high-density lipoprotein cholesterol; HOMA-IR = homeostasis model assessment of insulin resistance; hs-CRP = high-sensitive C-reactive protein.

### Unsupervised clustering for suspected CSA

As shown in Table 1 and Figure 1, k-means clustering of 20 standardized clinical and biochemical variables identified 2 distinct patient phenotypes. Cluster 0 (n = 231) was characterized by a metabolically adverse profile, including elevated levels of triglycerides, non–HDL-C, LDL-C, apoB, apoC2, apoC3, apoE, FFA, and HOMA-IR. In contrast, cluster 1 (n = 337) exhibited a lower metabolic burden but increased inflammatory activity, as reflected by the significantly higher hs-CRP and fibrinogen levels. Cluster 1 also had higher HDL-C and apoA1 levels, and current smoking, whereas cluster 0 showed a higher prevalence of hypertension. The reproducibility analysis of the clustering result remained at k = 2, and only 0.6% of patients changed cluster (Supplemental Figures 1 and 2). Furthermore, 16 out of the 20 variables were selected by least absolute shrinkage and selection operator. After checking for multicollinearity, the multivariable logistic regression model with 7 variables explained 92% of determination between cluster 0 and cluster 1 (Supplemental Table 2), with hs-CRP being one of the strongest determinants favoring cluster 1. The prevalence of CAS did not differ significantly between clusters (46.8% vs 39.8%; *P* = 0.12), and the distribution of spasm subtype (diffuse vs focal) did not differ significantly between clusters (diffuse spasm: 73 [67.6%] vs 100 [74.6%], focal spasm: 35 [32.4%] vs 34 [25.4%]; *P* = 0.23). Moreover, among the medications evaluated, only the use of CCBs differed significantly between the 2 clusters (*P* = 0.043), with higher usage in cluster 1. No significant differences were observed for other medications including angiotensin-converting enzyme inhibitors, angiotensin II receptor blockers, statins, aspirin, or beta-blockers (Table 1).

### Spasm-positive vs spasm-negative comparison within each cluster

As shown in Table 2, within cluster 0, CAS-positive patients had significantly higher levels of apoC2, apoC3, and triglycerides and lower levels of HDL-C and apoA1 than CAS-negative patients. The levels of inflammatory markers did not differ significantly between the groups. In cluster 1, HDL-C levels were significantly lower in the CAS-positive group, whereas other markers, including CRP and fibrinogen, were modestly higher than those in the CAS-negative group. Moreover, as shown in Table 3, multivariable logistic regression analysis revealed distinct cluster-specific determinants of coronary spasm. In cluster 0, lower HDL-C was independently associated with coronary spasm, whereas inflammatory markers were not significantly associated with spasm susceptibility. In cluster 1, lower HDL-C remained independently associated with coronary spasm and elevated hs-CRP was additionally identified as an independent determinant of spasm. Adjustment for CCB use did not materially alter the associations observed in either cluster. Lower HDL-C remained independently associated with coronary spasm in cluster 0 (OR: 0.97; 95% CI: 0.95-0.99; *P* = 0.010), whereas both lower HDL-C (OR: 0.98; 95% CI: 0.96-0.99; *P* = 0.036) and elevated hs-CRP (OR: 2.50; 95% CI: 1.05-5.92; *P* = 0.038) remained independent determinants in cluster 1.

**Table 2:** Comparisons of Clinical Parameters Between Patients With and Without Coronary Spasm Within Cluster Group

	Cluster 0 (N = 231)			Cluster 1 (N = 337)		
	Positive (n = 108)	Negative (n = 123)	*P* value	Positive (n = 134)	Negative (n = 203)	*P* value
Age (y)	63 [56-71]	66 [56-72]	0.44	69 [60-75]	67 [59-74]	0.11
Male (%)	60 (55.6%)	59 (48.0%)	0.25	79 (59.0)	106 (52.2)	0.22
Smoking (%)	42 (38.9%)	41 (33.3%)	0.38	67 (50.0)	94 (46.3)	0.51
HTN (%)	60 (55.6%)	76 (61.8%)	0.34	65 (48.5)	95 (46.8)	0.76
BMI (kg/m^2^)	24.1 [22.3-26.5]	24.0 [22.3-25.8]	0.46	23 [21.3-24.5]	23.1 [21.0-25.4]	0.62
LDL (mg/dL)	140 [123-158]	139 [121-159]	0.78	103 [86-121]	100 [84-119]	0.33
Triglyceride (mg/dL)	172 [130-235]	149 [113-215]	0.011	101 [78-126]	95 [75-129]	0.58
Non-HDL-C (mg/dL)	173 [159-193]	172 [158-190]	0.38	123 [111-141]	120 [105-140]	0.19
HDL-C (mg/dL)	40 [36-50]	46 [39-53]	0.003	48 [41-56]	52 [43-61]	0.041
ApoA1 (mg/dL)	118 [107-139]	125 [114-141]	0.042	132 [119-147]	133 [119-147]	0.59
ApoA2 (mg/dL)	21 [18-24]	22 [19-25]	0.52	24 [22-27]	25 [22-28]	0.36
ApoB (mg/dL)	115 [107-127]	112 [101-123]	0.031	86 [74-96]	83 [72-94]	0.16
ApoC2 (mg/dL)	5.9 [4.9-7.3]	5.3 [4.4-6.5]	0.005	3.8 [2.9-4.7]	3.5 [2.6-4.4]	0.13
ApoC3 (mg/dL)	11.6 [9.5-14.4]	10.6 [9.0-12.4]	0.021	8.1 [6.7-9.3]	7.9 [6.6-9.4]	0.95
ApoE (mg/dL)	5.4 [4.8-6.1]	5.2 [4.5-6.0]	0.11	4.1 [3.4-4.6]	4.0 [3.4-4.50]	0.60
FFA (mg/dL)	502 [348-603]	448 [334-586]	0.14	405 [308-527]	410 [273-547]	0.65
HOMA-IR	1.66 [1.11-2.53]	1.67 [1.17-2.60]	0.71	1.19 [0.79-1.60]	1.33 [0.88-2.16]	0.12
A1c (%)	5.7 [5.3-6.1]	5.6 [5.4-6.1]	0.51	5.5 [5.2-6.0]	5.5 [5.2-6.1]	0.53
FIB (mg/dL)	276 [240-304]	276 [239-312]	0.47	342 [290-408]	328 [272-382]	0.049
hs-CRP (mg/dL)	0.1 [0.04-0.31]	0.1 [0.03-0.30]	0.31	0.30 [0.15-0.45]	0.21 [0.13-0.40]	0.011

Values are median [25th-75th percentiles] or n (%).

HTN = hypertension; other abbreviations as in Table 1.

**Table 3:** Multivariable Logistic Regression Analysis for Associated Factors With Coronary Spasm in Each Cluster

	Cluster 0 (N = 231)			Cluster 1 (N = 337)		
	OR	95% CI	*P* value	OR	95% CI	*P* value
HDL-C (mg/dL)	0.97	0.95-0.99	0.010	0.98	0.96-0.99	0.036
ApoC2 (mg/dL)	1.13	0.99-1.28	0.050	1.18	0.99-1.39	0.060
FFA (mg/dL)	1.00	1.00-1.002	0.15	Not selected		
hs-CRP (mg/dL)	2.60	0.74-9.18	0.14	2.50	1.05-5.92	0.038
HOMA-IR	Not selected			0.83	0.68-1.00	0.053

Abbreviations as in Table 1.

### Simplified HDL-C/hs-CRP–based stratification and spasm susceptibility

When patients were categorized according to the combination of HDL-C and hs-CRP levels, based on cutoff points of 47.5 mg/dL and 0.205 mg/dL, respectively, the distribution differed markedly between clusters (chi-square = 69.7; *P* < 0.001). Cluster 0 was predominantly characterized by low HDL-C with low hs-CRP, whereas cluster 1 showed a higher prevalence of high hs-CRP, either alone or in combination with low HDL-C (Supplemental Figure 3). Across these HDL-C/hs-CRP–defined categories, the prevalence of ACh-induced coronary spasm differed significantly (Figure 1). Patients with concomitantly low HDL-C and high hs-CRP exhibited the highest spasm positivity rates, whereas those with preserved HDL-C and low hs-CRP showed the lowest prevalence of coronary spasm. Intermediate spasm rates were observed in patients with isolated low HDL-C or isolated hs-CRP elevation.

### Subgroup analysis: endothelial function according to HDL-C/hs-CRP categories

**Figure 2:**
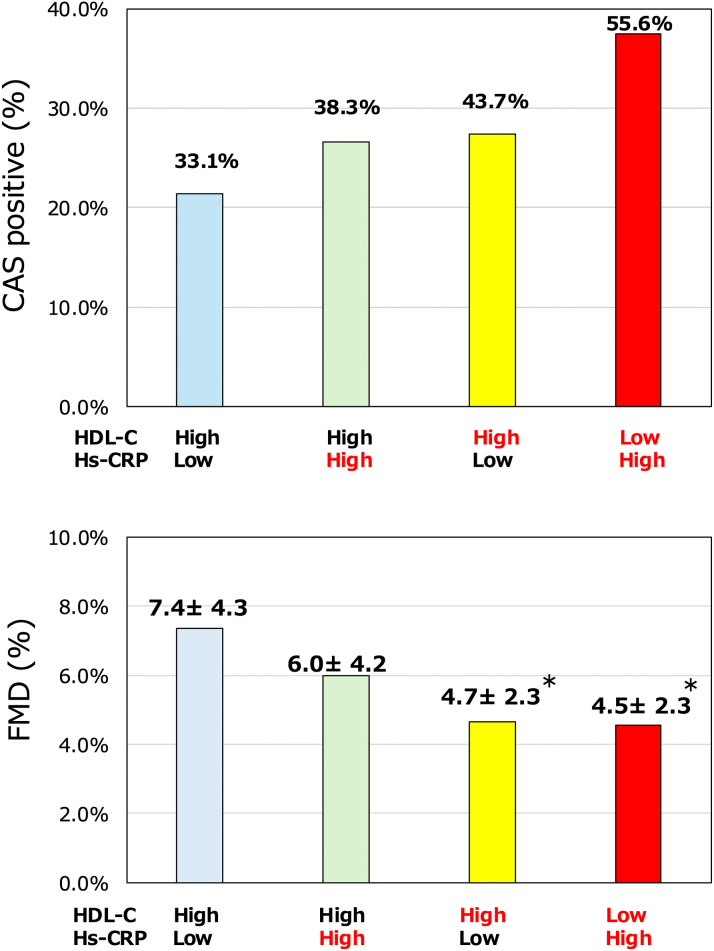
**Stratification by HDL-C and hs-CRP** This stratification was performed as a post hoc surrogate analysis to enhance clinical applicability, based on the results of the cluster-specific analyses. Upper panel: Prevalence of acetylcholine-induced coronary artery spasm in each quadrant defined by dichotomized HDL-C and hs-CRP (*P* = 0.001), representing metabolic and inflammatory burden, respectively. Lower panel: FMD values across the same HDL-C/hs-CRP–defined subgroups (*P* < 0.001). Data presented as percentage or mean (SD provided for FMD values). Groups compared using the chi-squared test (CAS positive) or ANOVA followed by Bonferroni post hoc test (FMD). ∗*P* < 0.01 vs high-HDL/low-CRP group. HDL-C = high-density lipoprotein cholesterol; hs-CRP = high-sensitivity C-reactive protein; CAS = coronary artery spasm; ANOVA = analysis of variance; other abbreviation as in Figure 1.

In the subset of 159 patients who underwent FMD testing, endothelial function differed significantly across the 4 groups defined by HDL-C and hs-CRP status (ANOVA, *P* < 0.001, Figure 2, lower panel). Patients with preserved HDL-C and low hs-CRP exhibited the highest FMD values, indicating relatively preserved endothelial function. In contrast, those with concomitantly low HDL-C and high hs-CRP showed the lowest FMD values, consistent with marked endothelial dysfunction. Intermediate FMD values were observed in patients with isolated low HDL-C or isolated hs-CRP elevation.

## Discussion

In this study, we applied unsupervised clustering combined with cluster-specific multivariable modeling to identify clinically meaningful phenotypes in patients with suspected CSA (Central Illustration). Two distinct phenotypes emerged: a metabolically dominant phenotype (cluster 0) characterized by global lipid derangement and insulin resistance, and an inflammation-dominant phenotype (cluster 1) with preserved lipid profiles but elevated systemic inflammation. Although the overall prevalence of ACh-induced CAS did not differ significantly between clusters, subgroup analysis revealed distinct biochemical patterns associated with spasm susceptibility within each phenotype. Moreover, using a simplified stratification based on 2 clinically accessible biomarkers, HDL-C as a surrogate of metabolic burden and hs-CRP as a marker of systemic inflammation, we demonstrated that patients with concomitant low HDL-C and high hs-CRP exhibited the highest prevalence of coronary spasm and the most severe endothelial dysfunction. This observation supports a synergistic interaction between metabolic and inflammatory stressors in CSA and highlights the feasibility of practical risk stratification without reliance on complex dimensionality reduction techniques.

**Central Illustration:**
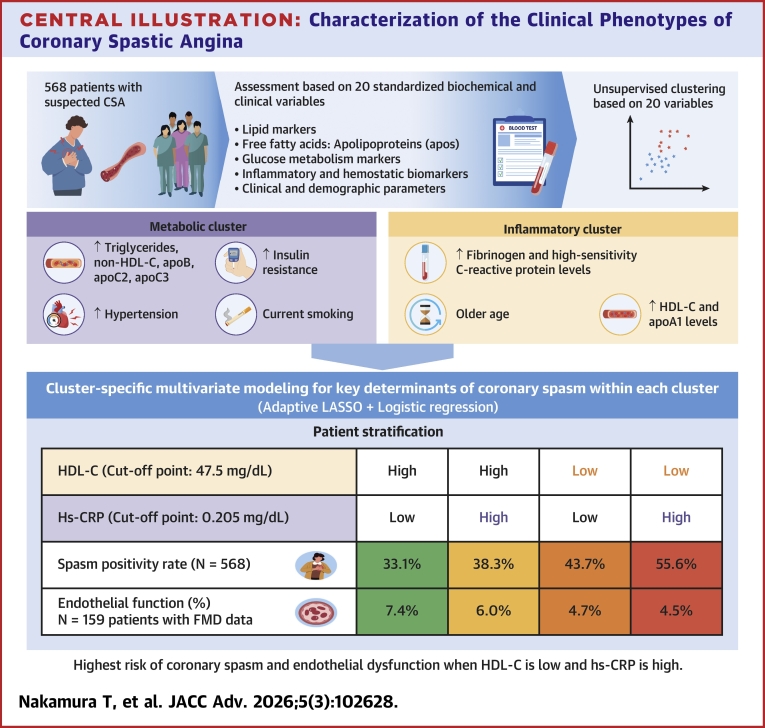
**Characterization of the Clinical Phenotypes of Coronary Spastic Angina** The “High” and “Low” labels refer to values above or below the respective cutoff points for HDL-C and hs-CRP. Colors other than black indicate abnormal findings. The unit of endothelial dysfunction is expressed as a percentage, not as a measure of prevalence. LASSO = least absolute shrinkage and selection operator; other abbreviations as in Figures 1 and 2.

These findings may help explain the persistent symptom burden observed in CSA, even with standard pharmacologic treatment. While long-acting CCBs are known to improve prognosis and reduce major adverse cardiac events, over 60% of patients continue to experience recurrent anginal attacks during follow-up.^1^^,^^2^^,^^9^ Persistent symptoms contribute to impaired quality of life and substantial health care utilization, underscoring a major unmet need for improved risk stratification and individualized management strategies.^22^ Our phenotypic framework may offer mechanistic insight into this therapeutic gap by demonstrating that CAS occurs in the context of distinct underlying pathobiologies that may not respond equally to vasodilator therapy alone. Although some CCBs have pleiotropic properties, such as endothelial protection and anti-inflammatory effects,^23^ their impact on lipid metabolism and insulin resistance is limited. Therefore, symptom persistence in some patients may reflect insufficient targeting of underlying endothelial dysfunction or metabolic-inflammation axes. Our results suggest that phenotype-guided adjunctive therapies—such as statins, fibrates, insulin sensitizers, or anti-inflammatory agents—may improve treatment response in select populations. However, we did not directly assess therapeutic outcomes by phenotype, and this remains speculative. Therefore, future studies are warranted to test whether patients with a metabolically driven phenotype would benefit from adjunctive therapies targeting insulin resistance and dyslipidemia, and whether an inflammatory phenotype might respond to therapies modulating inflammation or Rho-kinase activity.^1^^,^^7^^,^^24^ Incorporating phenotype-based stratification into clinical decision-making could thus lead to more precise therapeutic selection, with the goal of improving symptom remission rates and long-term quality of life.^2^

Within cluster 0, CAS-positive individuals showed significantly higher triglycerides, apoC2, and apoC3, and lower HDL-C and apoA1 levels compared to CAS-negative individuals—consistent with atherogenic dyslipidemia and insulin resistance.^25^^,^^26^ These abnormalities likely impair nitric oxide bioavailability and promote endothelial dysfunction. In cluster 1, CAS-positive patients exhibited modestly higher hs-CRP and fibrinogen levels, alongside lower HDL-C, suggesting that smooth muscle hypercontractility driven by systemic inflammation may play a larger role—potentially via enhanced Rho-kinase activity and heightened sympathetic tone.^16^^,^^24^ An important finding of the present study is that reduced HDL-C emerged as a common determinant of coronary spasm susceptibility across both metabolic- and inflammation-dominant phenotypes. Although cluster 1 was characterized by relatively preserved lipid profiles at the group level, lower HDL-C within this cluster was still associated with a higher likelihood of coronary spasm, indicating that HDL-C represents a fundamental axis of vascular vulnerability that transcends phenotypic classification. HDL-C plays a central role in vascular protection through multiple mechanisms, including enhancement of endothelial nitric oxide bioavailability, antioxidative effects, and modulation of endothelial inflammation. Consequently, even modest reductions in HDL-C may impair endothelial resilience and lower the threshold for vasomotor dysfunction. In this context, HDL-C may function not merely as a lipid marker but as an integrated indicator of endothelial health.

The consistent association between low HDL-C and coronary spasm across phenotypes also supports the clinical utility of HDL-C as a simplified surrogate marker. While metabolic and inflammatory pathways contribute heterogeneously to coronary spasm pathophysiology, impaired HDL-mediated vascular protection appears to represent a shared downstream mechanism. This may explain why HDL-C retained predictive relevance even in patients without overt metabolic derangement and underscores its value in phenotype-guided risk stratification.

Moreover, even in patients with higher HDL-C and lower hs-CRP representing a metabolically and inflammatory favorable profile, approximately one-third experienced coronary spasm. This observation underscores the fact that conventional risk factor assessment alone is insufficient to identify all patients at risk. Additional contributors, including hormonal fluctuations, autonomic dysregulation, or genetic polymorphisms affecting endothelial or smooth muscle function, may underlie susceptibility in these individuals.^27^, ^28^, ^29^ Future research integrating genetic and multiomics data may enable more comprehensive phenotyping.

Our analysis also highlighted the differential contributions of metabolic and inflammatory factors to endothelial dysfunction as assessed by FMD. Lower HDL-C levels, reflecting impaired metabolic vascular protection, appeared to exert a dominant influence on endothelial function, as evidenced by significantly reduced FMD in patients with low HDL-C regardless of inflammatory status. Systemic inflammation, as indicated by elevated hs-CRP, was associated with a trend toward further endothelial impairment when present concurrently with low HDL-C, although inflammation alone did not significantly reduce endothelial function. These findings support the concept that dual-pathway targeting of metabolic and inflammatory abnormalities may be required to optimize vascular health in high-risk patients. However, because FMD primarily reflects endothelium-dependent vasodilation, it may not capture smooth-muscle-mediated hyperreactivity.^30^^,^^31^ Complementary assessments, such as nitroglycerin-mediated dilation, intracoronary ACh provocation, and autonomic function testing, could provide further insights in future studies. Taken together, these findings suggest that hs-CRP reflects a predominantly smooth muscle oriented inflammatory vulnerability that amplifies vasomotor hyperreactivity, rather than serving as a primary determinant of endothelial dysfunction.^32^

This study has several limitations. This was a single-center observational analysis, and external validation in independent cohorts is warranted. The number of patients with FMD assessment was limited, and direct evaluations of smooth muscle function and genetic predisposition were not performed. In addition, because the study population was restricted to patients without significant obstructive coronary artery disease, the generalizability of the findings to patients with more advanced atherosclerotic burden may be limited. This selection may also have attenuated potential differences in angiographic spasm patterns, such as focal vs diffuse spasm, which have been reported to be influenced by localized atherosclerotic lesions. Taken together, these limitations should be considered when interpreting the present findings, which nonetheless provide a pathophysiological framework for understanding the heterogeneous mechanisms underlying CSA and for informing future phenotype-oriented investigations.

## Conclusions

We identified distinct metabolic and inflammatory phenotypes within the CSA, each linked to specific biochemical signatures, variable endothelial dysfunction, and differential susceptibility to coronary spasm. The coexistence of high metabolic and inflammatory burdens confers the greatest risk of vascular injury and persistent symptoms. Notably, a simple combination of HDL-C and hs-CRP effectively captures this high-risk phenotype, providing a clinically accessible framework for risk stratification. Incorporating phenotype-based assessments into routine clinical practice may improve risk stratification, enable more individualized treatment approaches, and ultimately address the critical unmet need for more effective symptom control in patients with CSA.

Perspectives **COMPETENCY IN PATIENT CARE:** Phenotypic heterogeneity in CSA identified using metabolic and inflammatory profiling, highlighting implications for personalized management strategies. A simplified stratification using HDL-C and hs-CRP identified patients at highest risk of coronary spasm and endothelial dysfunction, as assessed by FMD, providing a clinically accessible framework for individualized risk assessment and management.

**TRANSLATIONAL OUTLOOK:** External validation in independent and more diverse cohorts is needed to confirm the generalizability of these findings. Future research should incorporate direct assessments of vascular smooth muscle function and genetic predisposition to better characterize the mechanisms of CSA. Broader inclusion criteria may also help clarify the clinical relevance of angiographic spasm patterns across varying stages of coronary artery disease.

### Data Sharing Statement

Data generated or analyzed during this study are available from the corresponding author upon reasonable request.

## Funding support and author disclosures

This study was supported by the Grant-in-Aid for Scientific Research (21K07387) from the 10.13039/501100001691Japan Society for the Promotion of Science (Tokyo, Japan). The authors have reported that they have no relationships relevant to the contents of this paper to disclose.
