# Supplementary material for: Unveiling Phenotypic Heterogeneity in Coronary Spastic Angina Through Multidimensional Risk Profiling: The FUJI-SPASM Study
Source: JACC Adv. 2026 Mar 25;5(3):102628. doi: 10.1016/j.jacadv.2026.102628 (PMC13352000; doi:10.1016/j.jacadv.2026.102628)
Supplement: Supplemental_Material [file mmc1.docx]

**Supplemental Tables**

**Supplemental Table 1. Comparisons of clinical parameters between patients with and without FMD measurements**

|  | Without FMD measurements  (N = 409) | With FMD measurements  (N = 159) | P-value |
| --- | --- | --- | --- |
| Age (yrs) | 67.0 [58.0-74.0] | 66.0 [57.0-72.0] | 0.14 |
| Sex, male, n (%) | 217 (53.1) | 87 (54.7) | 0.72 |
| Smoking, n (%) | 180 (44.0) | 161 (47.8) | 0.005 |
| Hypertension, n (%) | 136 (58.9) | 64 (40.3) | 0.42 |
| BMI (kg/m^2^) | 23.2 [21.4-25.5] | 23.7 [22.0-25.7] | 0.059 |
| LDL-C (mg/dL) | 116 [92-134] | 115 [93-143] | 0.63 |
| Triglyceride (mg/dL) | 118 [86-160] | 126 [92-175] | 0.11 |
| HDL-C (mg/dL) | 47 [40-57] | 48 [40-59] | 0.58 |
| Non-HDL-C (mg/dL) | 141 [116-167] | 142 [118-173] | 0.20 |
| ApoA1 (mg/dL) | 129 [115-145] | 128 [111-145] | 0.54 |
| ApoA2 (mg/dL) | 24 [20-27] | 23 [20-27] | 0.56 |
| ApoB (mg/dL) | 94 [79-111] | 96 [81-115] | 0.22 |
| ApoC2 (mg/dL) | 4.3 [3.2-5.5] | 4.5 [3.3-5.8] | 0.17 |
| ApoC3 (mg/dL) | 9.0 [7.3-11.0] | 8.9 [7.6-11.5] | 0.19 |
| ApoE (mg/dL) | 4.4 [3.7-5.3] | 4.6 [3.7-5.4] | 0.92 |
| FFA (mg/dL) | 441 [313-566] | 421 [311-556] | 0.54 |
| HOMA-IR | 1.38 [0.96-2.10] | 1.42 [0.95-2.44] | 0.51 |
| HbA1c (%) | 5.6 [5.2-6.0] | 5.5 [5.3-6.1] | 0.40 |
| FIB (mg/dL) | 305 [265-368] | 290 [258-341] | 0.082 |
| hsCRP (mg/dL) | 0.20 [0.12-0.40] | 0.20 [0.11-0.32] | 0.39 |
| **Medication use, n (%)** |  |  |  |
| ACE-I / ARB | 59 (14.4) | 28 (17.6) | 0.34 |
| CCB | 56 (13.7) | 29 (18.2) | 0.17 |
| Statin | 83 (20.3) | 37 (23.3) | 0.44 |
| Aspirin | 43 (10.5) | 22 (13.8) | 0.26 |
| Beta blocker | 6 (1.5) | 6 (3.8) | 0.086 |
| Coronary spasm, n (%) | 179 (43.8) | 63 (39.6) | 0.37 |

Abbreviations as in Table 1.

**Supplemental Table 2. Multivariable logistic regression analysis to identify factors associated with each cluster**

|  | Odds ratio | 95% CI | P value |
| --- | --- | --- | --- |
| **Favor Cluster 0** |  |  |  |
| ApoC2 (per mg/dL) | 0.461 | 0.370–0.574 | < 0.001 |
| HOMA-IR (per 1) | 0.808 | 0.694–0.940 | 0.006 |
| TG (per mg/dL) | 0.982 | 0.976–0.988 | < 0.001 |
| FFA (per mg/dL) | 0.999 | 0.998–0.999 | 0.047 |
| **Favor Cluster 1** |  |  |  |
| Smoking (vs. no smoking) | 2.028 | 1.209–3.399 | 0.007 |
| hs-CRP (per mg/dL) | 20.304 | 6.052–68.113 | < 0.001 |

Cluster 1 was coded as the outcome in the binary logistic regression model.

Abbreviations as in Table 1.

**Supplemental Figure 1. Silhouette analysis plots**

Panel (A): original cohort; Panel (B): validation cohort, after excluding 23 outliers in original cohort. The values on the y-axis represent the average silhouette coefficient for each specific cluster.

**Supplemental Figure 2. Principal component analysis and outlier detection in the validation cohort**

Panel (A): Scree plot derived from the principal component analysis of the validation data (N = 545); Panel (B): Scatter plot illustrating the distribution of 23 outliers (blue dots) identified within the total population (N = 568)


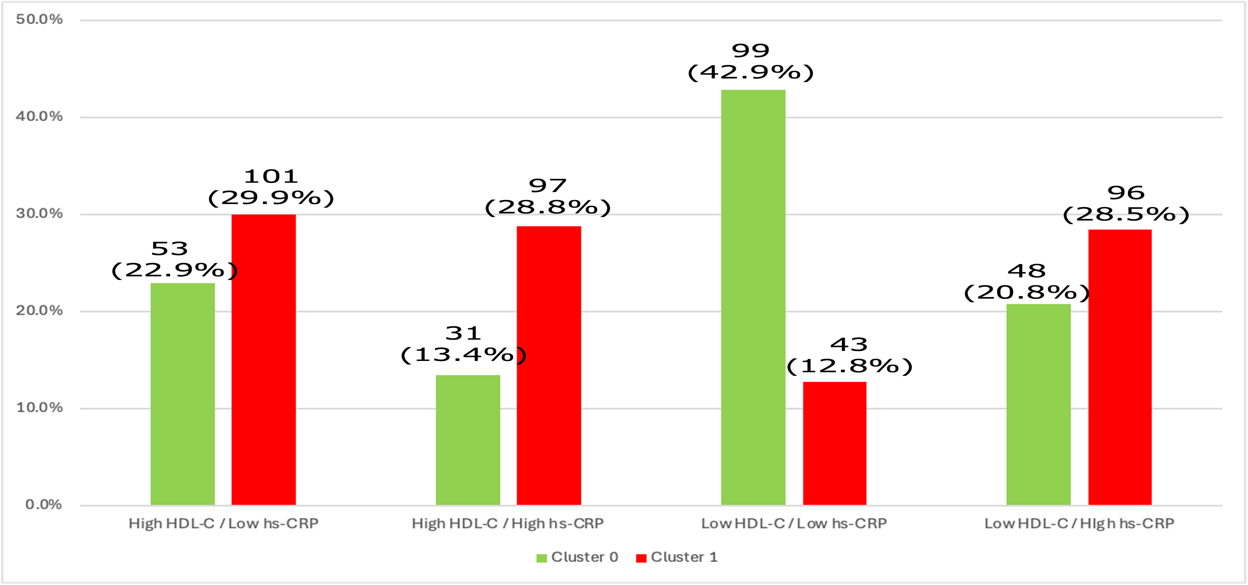
**Supplemental Figure 3. Distribution of patients across HDL-C and hs-CRP defined categories by Cluster**

When patients were categorized according to the combination of HDL-C and hs-CRP levels, the distribution differed markedly between clusters (χ² = 69.7, p < 0.001). Cluster 0 was predominantly characterized by low HDL-C with low hs-CRP, whereas Cluster 1 showed a higher prevalence of elevated hs-CRP, either alone or in combination with low HDL-C
